# *ANGUSTIFOLIA* is a central component of tissue morphogenesis mediated by the atypical receptor-like kinase STRUBBELIG

**DOI:** 10.1186/1471-2229-13-16

**Published:** 2013-01-31

**Authors:** Yang Bai, Prasad Vaddepalli, Lynette Fulton, Hemal Bhasin, Martin Hülskamp, Kay Schneitz

**Affiliations:** 1 Botanisches Institut III, Universität Köln, Zülpicher Straße 47b, 50674, Köln, Germany; 2 Entwicklungsbiologie der Pflanzen, Wissenschaftszentrum Weihenstephan, Technische Universität München, Emil-Ramann-Str. 4, 85354, Freising, Germany; 3 Present address: Department of Plant Microbe Interactions, Max Planck Institute for Plant Breeding Research, Carl-von-Linné-Weg 10, 50829, Köln, Germany; 4 Present address: School of Biological Sciences, Monash University, 3800, Melbourne, VIC, Australia

**Keywords:** ANGUSTIFOLIA, Flower, Ovule, Receptor-like kinase, Root hair patterning, Signal transduction, Tissue morphogenesis, STRUBBELIG

## Abstract

**Background:**

During plant tissue morphogenesis cells have to coordinate their behavior to allow the generation of the size, shape and cellular patterns that distinguish an organ. Despite impressive progress the underlying signaling pathways remain largely unexplored. In *Arabidopsis thaliana*, the atypical leucine-rich repeat receptor-like kinase STRUBBELIG (SUB) is involved in signal transduction in several developmental processes including the formation of carpels, petals, ovules and root hair patterning. The three *STRUBBELIG-LIKE MUTANT* (*SLM*) genes *DETORQUEO* (*DOQ*), *QUIRKY* (*QKY*) and *ZERZAUST* (*ZET*) are considered central elements of *SUB*-mediated signal transduction pathways as corresponding mutants share most phenotypic aspects with *sub* mutants.

**Results:**

Here we show that *DOQ* corresponds to the previously identified *ANGUSTIFOLIA* gene. The genetic analysis revealed that the *doq-1* mutant exhibits all additional mutant phenotypes and conversely that other *an* alleles show the *slm* phenotypes. We further provide evidence that SUB and AN physically interact and that AN is not required for subcellular localization of SUB.

**Conclusions:**

Our data suggest that *AN* is involved in *SUB* signal transduction pathways. In addition, they reveal previously unreported functions of *AN* in several biological processes, such as ovule development, cell morphogenesis in floral meristems, and root hair patterning. Finally, SUB and AN may directly interact at the plasma membrane to mediate SUB-dependent signaling.

## Background

Tissue morphogenesis and cellular patterning require extensive cellular communication. In plants the coordination of cellular behavior within a tissue is intrinsically linked to cell wall biogenesis and dynamics, as plant cells are connected through semi-rigid cell walls that drastically limit their relative movement. It is a major current challenge in plant biology to understand the mechanistic basis of intercellular communication and its connection to the cell wall during tissue morphogenesis.

In Arabidopsis, intercellular signaling mediated by the atypical leucine-rich repeat transmembrane receptor-like (LRR-RLK) STRUBBELIG (SUB) is essential for a number of developmental processes [1-6]. Ovules of *sub* mutants show frequent defects in the initiation and outgrowth of the outer integument. In addition, *sub* mutants exhibit twisted stems, petals and carpels/siliques. These phenotypes indicate a role for *SUB* in the control of integument initiation and outgrowth as well as stem and floral organ shape [1,2,6]. *SUB* also plays a role in internode length (and thus stem height), a trait that is potentially important for optimizing yield in crop plants.

At the cellular level, frequent misorientations of cell division planes were observed in e.g. L1 and L2 cells of young apical and floral meristems of *sub* mutants. Therefore, it was postulated that *SUB* signaling plays a role in orienting cell division planes in initiating integuments and floral meristems and thus influences the morphogenetic behavior of cells in a tissue context [1]. In addition, *SUB*, also known as *SCRAMBLED* (*SCM*), is involved in root hair patterning [3,4]. In this context, *sub* mutations lead to a randomization of root hair patterning such that root hairs develop ectopically or are not formed in the correct files.

In accordance with a perceived role of *SUB* in coordinating cellular behavior in tissue morphogenesis and cell patterning, *SUB* acts in a non-cell-autonomous fashion and mediates inter-cell-layer signaling across histogenic cell layers in the ovule, the floral meristem [5] and the root [7].

SUB belongs to the LRRV/STRUBBELIG-RECEPTOR FAMILY (SRF) family [8,9] and has several protein domains including an extracellular domain with seven leucine-rich repeats, a transmembrane domain and a cytoplasmic putative kinase domain [1,3,6]. Interestingly, a set of biochemical and genetic data indicated that although the kinase domain is essential for SUB function, enzymatic phosphotransfer activity is not [1,6]. Thus, SUB is likely a so-called atypical or dead kinase.

Signaling by atypical kinases is poorly understood in plants [10,11]. In addition, a detailed structure-function analysis of *SUB* suggested that the organ or cell-specific aspects of SUB-mediated signaling are not integrated at the SUB receptor, but involve other components that act together with, or downstream of SUB [6]. In order to unravel the signal-transduction pathway of *SUB* we have previously identified three complementation groups sharing the *sub*-like mutant (*slm*) phenotypes [2]. In addition, it was found that there is significant overlap in *SLM*-sensitive gene expression. Taken together the results indicated that *SLM* genes contribute to *SUB* signal transduction. The corresponding genes are called *QUIRKY* (*QKY*), *ZERZAUST* (*ZET*), and *DETORQUEO* (*DOQ*) [2]. Initial molecular analysis suggested that *QKY* encodes a putative membrane-localized protein with four C2 domains thus potentially connecting SUB to membrane-associated Ca^2+^- and phospholipid-dependent signaling [2].

In this work we focused on the *DOQ* gene. We show that *doq-1* is a mutant allele of the *ANGUSTIFOLIA* (*AN*) gene. The *doq-1* mutant carries a point mutation in the *AN* gene and we further demonstrate that *doq-1* shares phenotypes with other *an* alleles and conversely, other *an* alleles show all *slm* phenotypes tested. These results rule out the possibility that *doq-1* is an atypical allele. In addition, we provide evidence that SUB and AN can physically interact and that *AN* does not influence subcellular SUB distribution. Together our results reveal that *AN* is involved in *SUB*-dependent signaling events.

## Results

### *doq-1* mutants exhibit an underbranched trichome and narrow leaf phenotype

**Figure 1:**
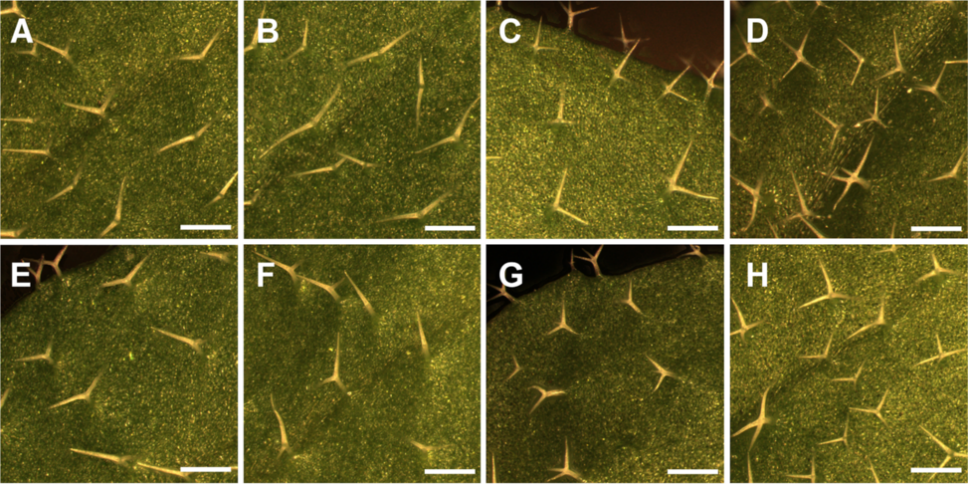
**Trichome branching in WT, *an *mutants and rescued lines.** Trichomes in the *an-1* mutant (**A**), the *an-2* mutant (**B**), the *an-2 35S::YFP:AN* #2 line (**C**), the *an-2 35S::YFP:AN* #4 line (**D**), the *doq-1* mutant (**E**), the *an-EM1* mutant (**F**), WT L*er* (**G**) and WT Col-0 (**H**). Scale bars: 400 μm.

**Figure 2:**
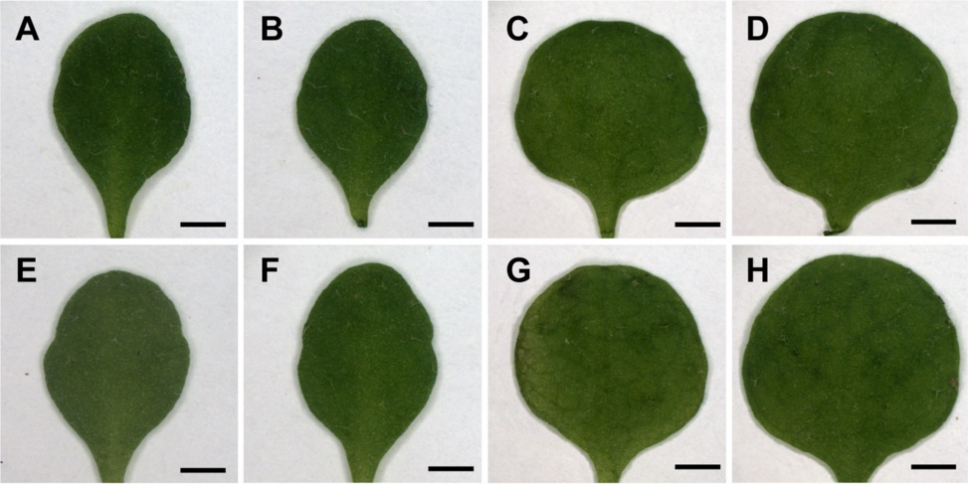
**Leaf shape in WT, *an *mutants and rescued lines.** The first pair of rosette leaves in the *an-1* mutant (**A**), the *an-2* mutant (**B**), the *an-2 35S::YFP:AN* #2 line (**C**), the *an-2 35S::YFP:AN* #4 line (**D**), the *doq-1* mutant (**E**), the *an-EM1* mutant (**F**), WT L*er* (**G**) and WT Col-0 (**H**). Scale bars: 1.5 mm.

Meiotic recombination mapping placed *DOQ* in a 330.6 kb interval at the top of chromosome 1 (see Methods). This interval included *AN*, a gene previously described to affect trichome branching, leaf morphology, and silique shape [12-15]. During the course of this analysis we noticed that *doq-1* trichomes are underbranched. Together with the previously described narrow leaf phenotype of *doq-1*[2], this suggested that *DOQ* and *AN* functions are related. We therefore compared the *doq-1* mutant leaf and trichome phenotype with three reference *an* alleles, *an-1*, *an-2*, *an-EM1* and two *an-2 35S::YFP:AN* rescued lines. In *doq-1* mutants, reductions in trichome branching approached levels seen in the *an-1*, *an-2*, *an-EM1* alleles (Figure 1, Table 1). Two-branched and three-branched trichomes are almost absent and a new class of unbranched trichomes was observed. The leaf shape of *doq-1* mutants was also similar to that seen in *an-1*, *an-2*, *an-EM1* mutants (Figure 2). Correspondingly and in step with the reference *an* alleles, the leaf length/width ratio in *doq-1* was significantly increased (Table 2).

**Table 1:** **Frequency of trichomes with different branch numbers in WT and *an *mutant lines on the fifth rosette leaves**

**Genotype**^**a**^	**unbranched**	**One branch**	**Two branches**	**Three branches**
L*er*	0	2.8	95.2	2.0
Col-0	0	1.7	83.3	15.0
*an-EM1*	1.9	96.2	1.9	0
*doq-1*	2.0	97.0	1.0	0
*an-1*	4.8	95.2	0	0
*an-2*	3.6	95.4	1.0	0
*an-2 35S::YFP:AN #2*	0	3.5	87.7	8.8
*an-2 35S::YFP:AN#4*	0	2.4	88.0	9.6

^a^n = 300 trichomes for each genotype.

**Table 2:** Length and width measurements in rosette leaves

**Genotype**	**Ratio (L/W)**^**a,b,c**^
L*er*	1.08 (±0.06)
Col-0	1.03 (±0.05)
*an-EM1*	1.46 (±0.07)
*doq-1*	1.38 (±0.08)
*an-1*	1.42 (±0.10)
*an-2*	1.47 (±0.08)
*an-2 35S::YFP:AN #2*	1.03 (±0.07)
*an-2 35S::YFP:AN#4*	1.07 (±0.04)

^a^The value was calculated from 20 rosette leaves in each lines.

^b^The ratios are calculated by the formula: ratio=length (L)/width (W). Values represent mean ± SD.

^c^Two-way ANOVA followed by HSD Tukey test. Comparisons were done between individual mutant/wild-type pairs (p<0.001) and between individual *an-2 35S::YFP:AN*/wild-type pairs (p>0.05).

### The *DOQ* gene corresponds to the *AN* gene

We tested whether the *DOQ* gene corresponds to the *AN* gene. Genetic analysis revealed no complementation of *doq-1* with *an-1* indicating that *doq-1* is allelic to *an*. Furthermore, we sequenced the *AN* gene in the *doq-1* mutant and demonstrated a G to A transition at position 509 in the cDNA coding region. This mutation causes a glycine to aspartic acid substitution at position 170 that is located in the predicted NAD(P)-binding domain.

### *an* alleles show *slm* phenotypes

As most *slm* phenotypes had not been reported for *an* mutants, the question arose whether *doq-1* is an atypical *an* allele showing new phenotypes or whether all *an* alleles share the *slm* phenotypes. We therefore compared *slm* phenotypes between *doq-1*, the *an-1*, *an-2*, *an-EM1* alleles and *an-2 35S::YFP:AN* rescued lines.

**Figure 3:**
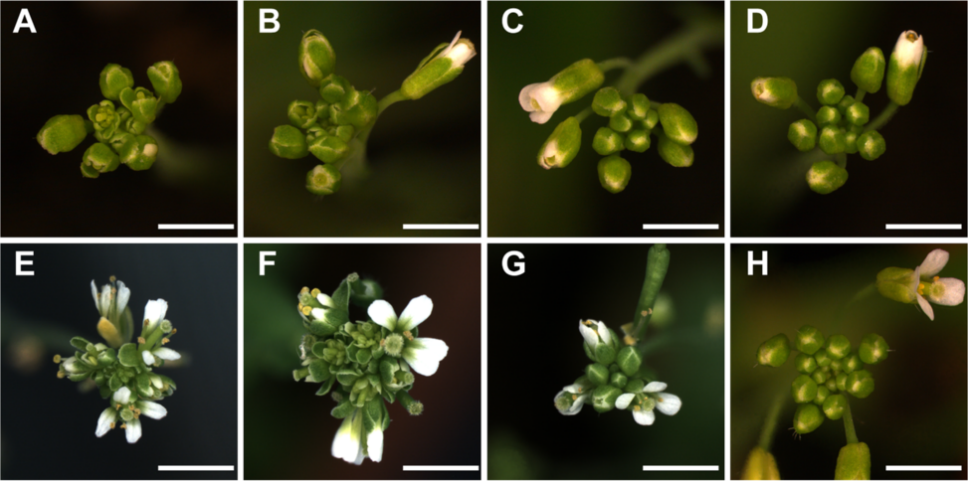
**Inflorescence in WT, *an *mutants and rescued lines.** Prematurely opened flower buds in the *an-1* mutant (**A**), the *an-2* mutant (**B**), the *doq-1* mutant (**E**) and the *an-EM1* mutant (**F**). Premature opening of flowers is rescued in the *an-2 35S::YFP:AN* #2 line (**C**) and *an-2 35S::YFP:AN* #4 line (**D**), as compared to WT L*er* (**G**) and WT Col-0 (**H**). Scale bars: 3 mm.

**Figure 4:**
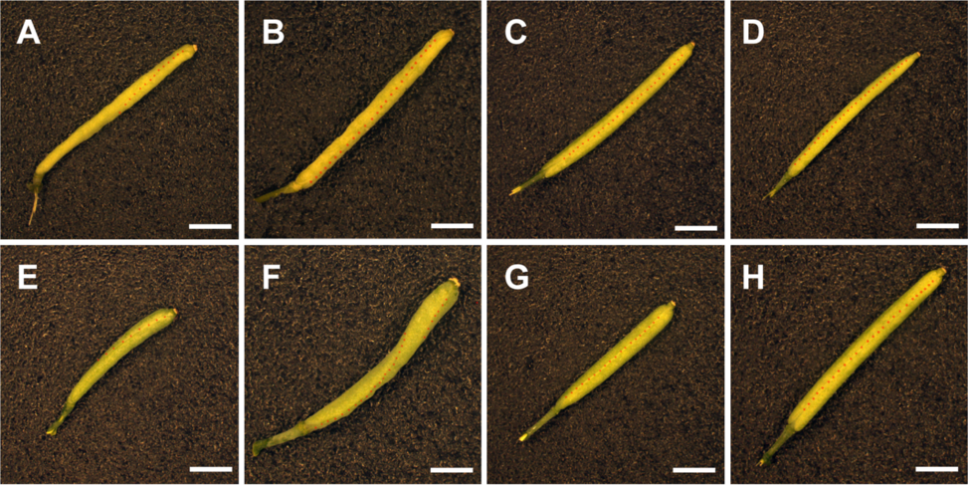
**Silique morphology in WT, *an *mutants and rescued lines.** Twisted siliques are evident in the *an-1* mutant (**A**), *an-2* mutant (**B**), *doq-1* mutant (**E**) and the *an-EM1* mutant (**F**). The *an-2 35S::YFP:AN* #2 line (**C**) and *an-2 35S::YFP:AN* #4 line (**D**) exhibit normal silique morphology, compared to WT L*er* (**G**) and WT Col-0 (**H**). The replums are highlighted by dashed lines to indicate where twisting is present. Scale bars: 2 mm.

**Figure 5:**
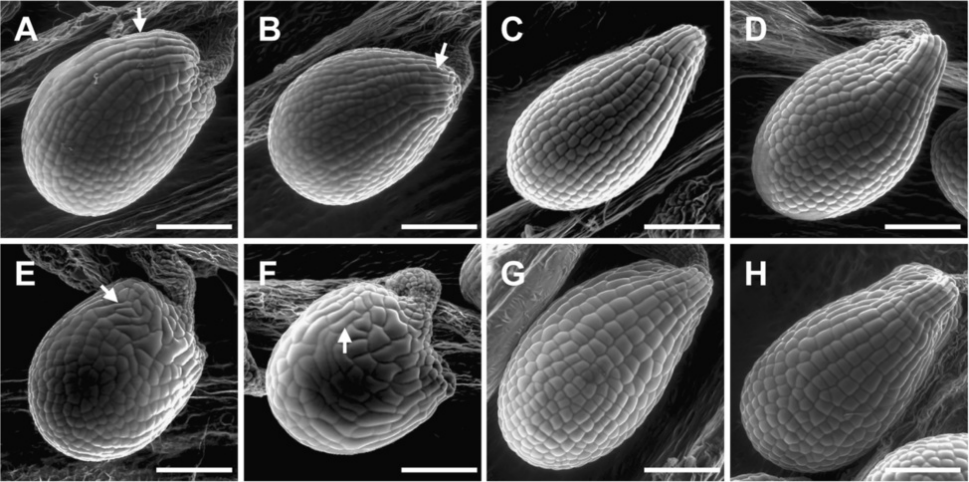
**Ovule shape in WT, *an *mutants and rescued lines.** Ovules in the *an-1* mutant (**A**), the *an-2* mutant (**B**), the *an-2 35S::YFP:AN* #2 line (**C**), the *an-2 35S::YFP:AN* #4 line (**D**), the *doq-1* mutant (**E**), the *an-EM1* mutant (**F**), WT L*er* (**G**) and WT Col-0 (**H**). Arrows point to regions with aberrant cell division. Scale bars: 100 μm.

**Figure 6:**
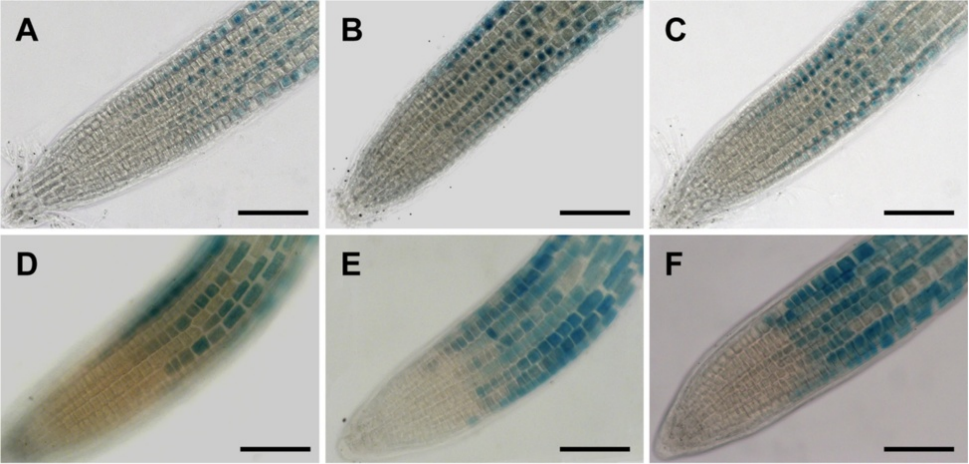
***GL2:GUS *expression pattern in WT and *an *mutants.** The expression pattern of the *GL2* promoter GUS fusion is shown in L*er* (**A**), the *doq-1* mutant (**B**), the *an-EM1* mutant (**C**), Col-0 (**D**), the *an-1* mutant (**E**), the *an-2* mutant (**F**), Scale bars: 100 μm.

As described for *doq-1,* all *an* alleles showed premature opening of flowers and twisted petals (Figure 3). These phenotypes are rescued in the *an-2 35S::YFP:AN* lines. In addition, *doq-1*-like twisting of siliques was observed in all *an* alleles and could be rescued by *AN* overexpression (Figure 4). Plants carrying the *doq-1* mutation show a weak ovule phenotype as compared to *sub* mutants [2]. To study the cellular patterns in *an* ovules we used Scanning Electron Microscopy. We observed smaller cells with atypical division planes at the distal end of the outer integument in all *an* alleles tested and a rescue of this phenotype in *an-2 35S::YFP:AN* lines (Figure 5). Finally, we analyzed the role of *AN* in root hair patterning. To this end we investigated the expression profiles of a GL2:GUS construct in *an-1*, *an-2*, *an-EM1* allelic backgrounds. GL2:GUS is expressed in atrichoblast cell files and serves as a convenient marker for root hair patterning [16]. Mutant *an-1*, *an-2*, *an-EM1* alleles displayed a severe distortion of the GL2:GUS pattern (Figure 6). Thus *AN* is a new component of the root hair patterning machinery.

### SUB and AN can interact directly

**Figure 7:**
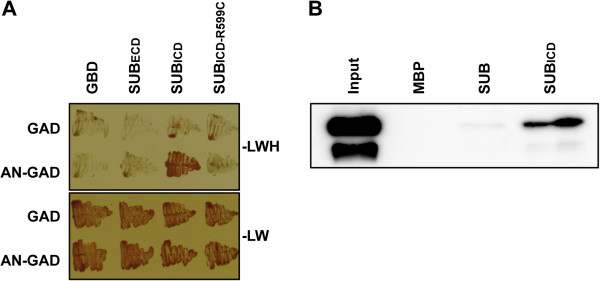
**SUB can physically interact with AN.** (**A**) Results of a yeast two-hybrid assay are depicted. The top panel shows that only cells carrying both plasmids encoding SUBICD and AN can grow on drop-out medium lacking leucine, tryptophan, and histidine (−LWH). Interaction depends on the presence of a functional SUBICD as indicated by the absence of signal in the variant SUBICD-R599C which mimics a strong *sub* mutation [6]. As a control all transformants can grow on medium lacking leucine and tryptophan (−LW) (bottom panel). Assays were done in the presence of 2.5 mM 3-amino-1,2,4-triazole (3-AT). GAD and GBD denote empty vector controls. (**B**) Western blot analysis of in vitro pull-down experiments to test MBP:SUB (full length) or MBP:SUBICD binding to GST:AN. AN was detected using a specific anti-AN antibody. The antibody detects two bands, both of which represent intact AN, as shown by MALDI-TOF analysis, suggesting two different conformations. GST:AN does not interact with MBP alone.

Next we addressed in more detail how *SUB* and *DOQ/AN* relate to each other during *SUB*-dependent signal transduction. Earlier results indicated that the two genes do not regulate each other at the transcriptional level [2]. We thus tested if DOQ/AN and SUB have the potential to interact directly at the protein level. Indeed, the intra-cellular domain of SUB (SUBICD, residues 371 to 768), but not the extra-cellular domain (SUBECD, residues 26 to 340), was able to interact with AN in a yeast two-hybrid assay (Figure 7). In addition, fusions of maltose-binding protein (MBP) to SUBICD and the SUB full-length proteins were able to interact with a fusion of glutathione-S-transferase (GST) to AN in in vitro pull-down assays involving bacterially-expressed recombinant proteins (Figure 7).

### *AN* is not required for subcellular localization of SUB:EGFP in roots

**Figure 8:**
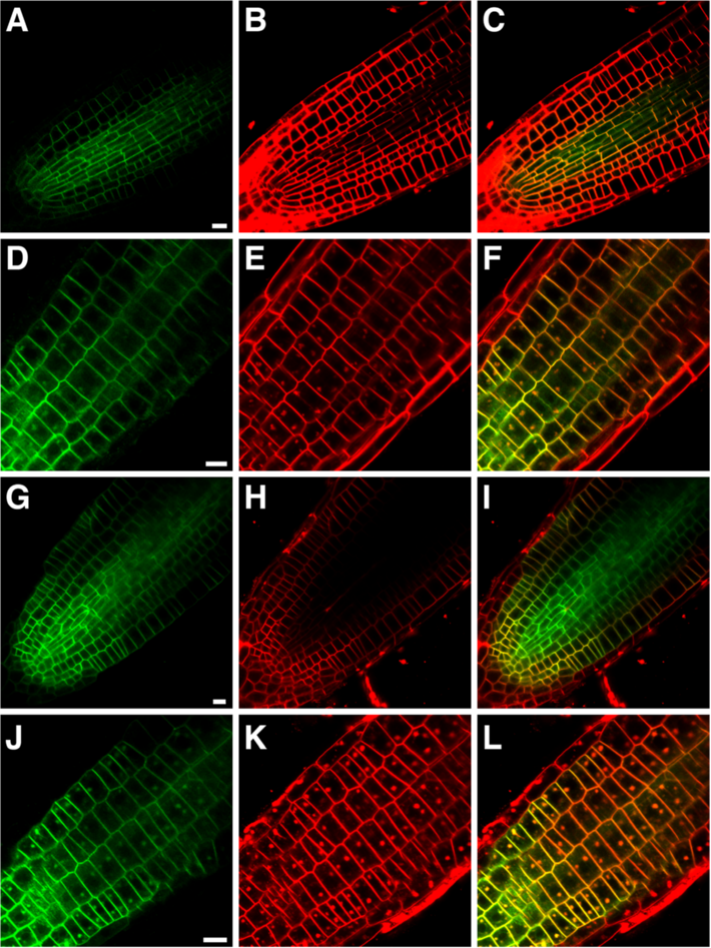
***SUB::SUB:EGFP *expression in five-days-old root tips of WT and *doq-1 *mutants in the absence and presence of BFA.** Confocal micrographs are shown. Two independent lines transgenic for a functional *SUB:SUB:EGFP* reporter [6] exhibiting a GFP-based signal were analyzed for each genotype. GFP-based signal is shown in (**A**, **D**, **G**, **J**), FM4-64-based counterstain in (**B**, **E**, **H**, **K**) and the overlay in (**C**, **F**, **I**, **L**). Mid-optical section through a wild-type root tip highlights the plasma membrane localization of SUB:EGFP (**A**-**C**). Tangential optical section through the epidermis of a wild-type root tip treated with 50 μm BFA for 30 minutes reveals the typical dotted structures that indicate the presence of BFA compartments (**D**-**F**). A similar set is shown for untreated *doq-1* roots (**G**-**I**) and BFA-treated *doq-1* roots (**J**-**L**). Scale bars: 10 μm.

Previous studies involving a functional SUB:EGFP fusion protein indicated that SUB is localized to the plasma membrane and undergoes brefeldin A (BFA)-sensitive recycling [5-7]. As the AN homolog in animals was identified as a Brefeldin A ribosylated substrate [17] we tested whether the subcellular localization of SUB is dependent on *DOQ/AN* function. Towards this end we analyzed expression of a functional *SUB::SUB:EGFP* reporter [6] in roots of *doq-1* mutants in the absence or presence of BFA (Figure 8). Expression of the reporter appeared normal under both experimental conditions indicating that *AN* is not required for correct subcellular localization and recycling of SUB.

### *ERECTA* influences the *an* phenotype

**Figure 9:**
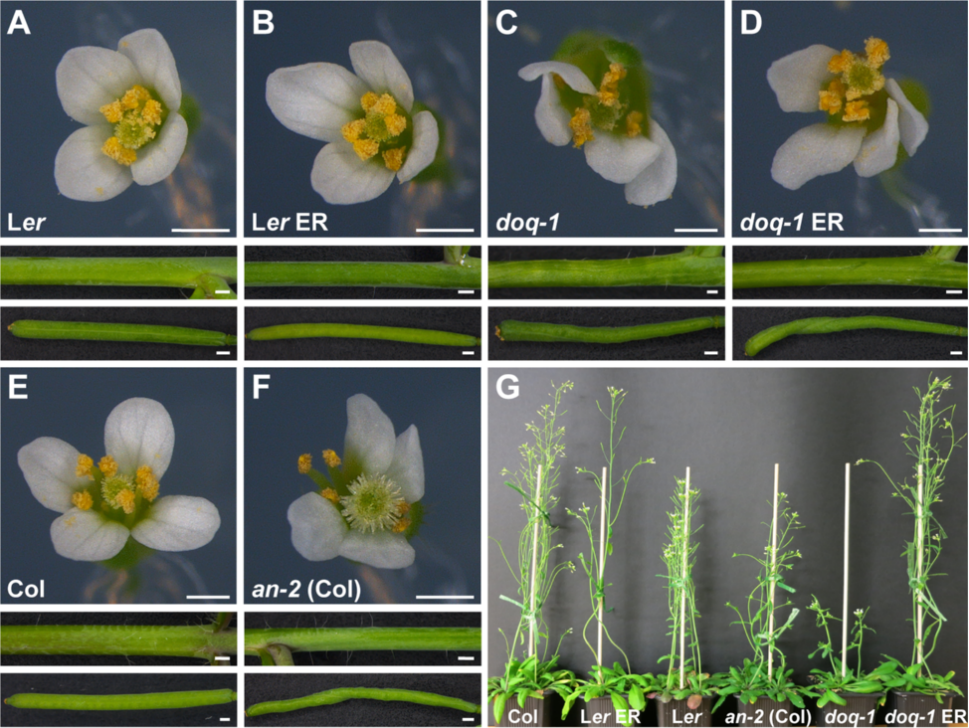
**The *doq-1 *above-ground phenotype in the presence of functional *ERECTA. *** Comparison of wild type, *an-2* (Col), *doq-1* (L*er*) and *doq-1* plants transgenic for Col *ER*-containing plasmid pKUT196. (**A**-**F**) Morphology of flowers (upper panel), stems (central panel) and siliques (bottom panel). (**A**) Wild-type L*er*. (**B**) Transgenic L*er* pKUT196. (**C**) *doq-1* mutant. Note the aberrant flower and silique morphology. Stem twisting is very mild if present at all. (**D**) Transgenic *doq-1* pKUT196. Note the irregular flower and silique morphology. Stem morphology is essentially normal (compare with F). (**E**) Col wild type. (**F**) Col *an-2* mutant. Note the aberrant flower and silique morphology. Stem twisting is nearly normal. (**G**) Plant height comparisons of six-week-old pKUT196 transgenic plants in comparison to wild type and mutant reference lines. Note the height reduction in *an-2* (Col) plants. Scale bars: 0.5 mm.

The *sub* phenotype is sensitive to the genetic background [1] and it was shown that mutations in *ERECTA* (*ER*), encoding a LRR-RLK [18], and *SUB* interact synergistically as *sub er* double mutants exhibit strongly reduced plant height [6]. To investigate this issue we transformed pKUT196, a plasmid containing 9.3 kb of Col-0 genomic DNA spanning the entire *ER* locus [18,19], into *doq-1* (L*er*) plants and asked whether a wild-type copy of *ER* would affect the *doq-1* phenotype. Interestingly, the transgene had a similar effect on plant height but not on other aspects of the *doq-1* and *sub-1* phenotypes (Figure 9) [6]. Similar to *sub-1 ER* plants, plant height was rescued in *doq-1 ER* plants indicating that height reduction in *doq-1* is caused by a synergistic interaction between the *doq-1* and *er* mutations. It should be noted, however, that plant height in *an-2* (Col) was slightly reduced as well, despite the presence of wild-type *ER*, though not to the extent as in *doq-1* (L*er*). This suggests that *AN* affects plant height in part independently of *ER*. Full rescue of plant height in *doq-1 ER* plants indicates the presence of additional modifiers that influence this trait in L*er*.

## Discussion

Multiple lines of evidence indicate that *AN* is involved in the *SUB*-dependent signaling mechanism. First, our genetic data show that all tested phenotypic aspects of *sub* are shared by *an* mutants. Second, *DOQ*/*AN* and *SUB* influence the expression of a common set of target genes. For example, 62 percent of genes misexpressed in *sub* flowers are also misexpressed in *doq-1*/*an*[2]. Thirdly, AN and SUB are able to interact physically in two different assays. Finally, *an* and *er* mutations synergistically affect internode length and plant height, as was observed for *sub* and *er*[6]. These results unexpectedly bring together two well-established but previously unconnected research fields. What do we learn for the function of *AN* and what for the function of *SUB*?

### *AN* is involved in a broad variety of cellular processes

*AN* was initially identified by its narrow leaf phenotype and trichome phenotype [14]. The closer analysis of the narrow leaf phenotypes suggested a role in cell polarity of leaf cells, such that the length of individual cells was increased and the width reduced [15,20]. As cell number is also changed an additional role in cell division control was postulated. The characterization of the trichome phenotype placed *AN* in a regulatory network controlling branching initiation [12,13]. In this context, a conspicuous lack of microtubule accumulation in the branch initiation zone of the developing trichome cell suggested a role of AN in the control of microtubule organization [21]. The new *slm* phenotypes of *an* mutants reported in this study indicate that *AN* is involved in a *SUB* dependent signal transduction cascade. This function is separate from the first two functional aspects derived from leaf shape and trichome phenotypes, as *sub* mutants do not share these phenotypic aspects [2]. Such a broad spectrum of functions is compatible with the proposed biochemical functions of the AN protein. The *AN* gene encodes a protein with homology to CtBP/BARS [21,22]. CtBPs (C-terminal Binding Protein) were initially discovered as proteins binding to the C-terminal domain of adenovirus EA-1 [23]. CtBPs have been described as co-repressors for many transcriptional repressors carrying a PxDLS or RRT protein motif [24,25]. A possible function of AN as a co-repressor is the finding that micro-array experiments revealed many genes that are transcriptionally regulated by AN [2,22]. CtBP/BARS were independently identified as Brefeldin A ADP ribosylated substrates (BARS) [17]. A possible Golgi-related function is supported by the finding that CtBP can induce constriction in Golgi tubules [26] and membrane fission [27]. In support of such a function AN was reported to act outside the nucleus [28].

### Role of *AN* in *SUB*-mediated signal transduction

How does *AN* fit into the *SUB*-mediated signaling pathway? *AN* is unlikely to be a direct or indirect transcriptional target of *SUB* signaling. For example, *SUB* expression is only minimally altered in *doq-1* flowers at various stages [2]. In addition, a *35S::SUB* transgene failed to rescue the phenotype of *doq-1* mutants indicating that *SUB* is not directly regulated at the transcriptional level by *AN*. At the same time, *AN* expression was not found to differ between floral tissue of wild type, *sub-1* and other *slm* mutants [2] (data not shown). These observations render it unlikely that *AN* and *SUB* regulate each other’s activity at the transcriptional level.

Subcellular localization of a functional SUB:EGFP fusion protein was found to be restricted to the plasma membrane [6,7]. A functional AN:GFP fusion protein was recently reported to reside in the cytoplasm and in punctate compartments around the trans-Golgi network (TGN) [28]. The TGN localization of AN led the authors to suggest a Golgi-related role for this protein, possibly in membrane trafficking. These results are compatible with at least two possibilities of how AN may fit into the SUB signaling mechanism. In the first scenario AN could mediate membrane trafficking of SUB, a view that is also indirectly supported by the finding that *QKY* encodes a putative membrane-localized protein thought to function in membrane-associated Ca^2+^- and phospholipid-dependent signaling [2]. However, this model does not fit the data presented in this study as signal distribution of a functional *SUB::SUB:EGFP* reporter was found to be unaltered in *doq-1* roots. In an alternative scenario, the cytoplasmic distribution of AN would allow its direct interaction with the intracellular domain of SUB.

We currently favor this notion as results from the yeast two-hybrid and in vitro pull-down assays suggest direct interaction between SUB and AN proteins at the plasma membrane. This interaction could then be necessary to control further downstream events of SUB signal transduction. AN is likely to mediate only some aspects of SUB signaling as there is a difference in the strength between for example the ovule phenotypes of *sub* and *doq-1* mutants and stem twisting is nearly absent in *doq-1* or *an-2* (Figure 9). Moreover, there is only partial overlap in misexpressed genes between *sub* and *doq-1* flowers [2]. With respect to plant height AN appears to be part of the SUB mechanism that interacts with the LRR-RLK ER. It was proposed that *ER* and *SUB* signaling converge either at the level of the receptors or at some level more downstream in the mechanism [6]. Thus, it will be interesting to resolve exactly how SUB, AN and ER relate to each other in future studies on SUB signaling.

## Conclusions

In this study we showed that phenotypes of the *slm* mutant *doq-1* and the trichome and leaf shape mutant *an* overlap. In addition, we showed that *doq-1* is allelic to *an*. We further demonstrated that *doq-1* is not an atypical *an* allele but that all tested *an* mutants show previously undescribed *slm* aspects. The data reveal a broader range of biological functions for *AN* than previously appreciated. Finally, our data reveal the possibility that SUB and AN interact directly. Taken together, the presented evidence suggests a role for AN in tissue morphogenesis mediated by the atypical receptor-like kinase SUB.

## Methods

### Plant work and genetics

The following *Arabidopsis thaliana* (L.) Heynh. lines were used in this study: Columbia (Col-0) and Landsberg (*erecta* mutant) (L*er*) wild-type strains, *doq-1*[2], *an-1*, *an-*2 [22], *an-EM1*[21], *an-2 35S::YFP:AN*. *an-2 35S::YFP:AN* plants were generated by cloning the CDS of the YFP:AN fusion into the pPAMPAT vector containing the 35S promoter (GenBank accession AY027531). Plasmid pKUT196 was described previously [19]. Plant transformation was performed according to the floral dip method [29].

Plants were grown on soil at 24°C with 16 hours of light per day. The *GL2:GUS* line (L*er*) [16] was introduced into *an* mutants by backcrosses. For GUS assays, plants were grown on MS plates for 4 days.

Using a L*er*/Col mapping population *doq-1* was localized to the upper end of chromosome 1 between markers F10O3(481D) and NF21M12 [2]. Further mapping revealed an interval of 330.6 kb. The final Northern marker 96_(BccI) was located at chromosomal position 96771 (one recombinant left). 96_(BccI) is a CAPS marker which yields the following products after PCR with primers 96_(BccI)_F (GGGCTTTGATTTGATTGTGG) and 96_(BccI)_R (AAGAGAGGAGTGCAGCCAAA) and BccI digestion: Ler - 498 bp, Col - 254, 244 bp. The final Southern marker was NT7123 (chromosomal position: 427,343 bp, 3 recombinants left). NT7123 is a SSLP marker that, following PCR with primers NT7123_F (GTGTCCTTTTTTCTCAACGATG) and NT7123_R (CATGCACGTACGATTTGTTTAAC), yielded the following products on a 3.5% agarose gel: Ler , <199 bp; Col, 199 bp.

### Yeast two-hybrid assay

The Matchmaker yeast two-hybrid system (Clontech) was employed and experimental procedures followed the manufacturer’s recommendations. The pACT-AN construct was described previously [21]. For the generation of pGBKT7-SUBICD, the intracellular part of SUB coding sequence [1] was amplified from cDNA using primers SUBintra_F (CATGCCATGGATAACCGATATTACAGTG) and SUBintra_R (ATCGGTCGACAATAAACTATTGCTTCTG). The PCR product was digested with NcoI/SalI and cloned into NcoI/SalI digested pGBKT7. The R599C mutation was introduced into pGBKT7-SUBICD by the QuikChange II XL site-directed mutagenesis kit according to the manufacturer’s recommendations (Agilent Technologies) by using primers 35SsubR599Cf (AAGAAGCTCACTTGGAATGTATGTATAAATATTGCATTAGGAGCTTC) and 35SsubR599Cr (GAAGCTCCTAATGCAATATTTATACATACATTCCAAGTGAGCTTCTT). For the cloning of pGBKT7-SUBECD, the extracellular part of SUB coding sequence was amplified from cDNA using primers SUB Extra/Nde1_F (GCTCATATGACTAATCTACGAGATGTTTCGGCGA and SUB Extra/Xma1_R (TACCCGGGGTTGAGTGGACCAGAATTTTCCTGATC). The PCR product was digested with NdeI/XmaI and cloned into NdeI/XmaI digested pGBKT7. All PCR-based constructs were sequenced.

To assay possible interactions in yeast pGBKT7 plasmids containing SUBECD, SUBICD and SUBICD-R599C were cotransformed with pACT or pACT-AN into yeast strain AH109. Transformants were selected after 3 days on SC medium lacking Leu and Trp (−LW) at 30°C. To examine yeast two-hybrid interactions, the transformants were grown on solid SC medium lacking Leu and Trp (SC-LW) or Leu, Trp, and His (SC-LWH) at 30°C.

### Generation of constructs for recombinant protein production

*AN* and *SUB* cDNA were cloned into gateway entry vector pDONR 201. The following primers were used for gateway cloning: for amplifying *AN* cDNA: GGGGACAAGTTTGTACAAAAAAGCAGGCTTCATGAGCAAGATCCGTTCG and GGGGACCACTTTGTACAAGAAAGCTGGGTCTTAATCGATCCAACGTGTGATAC; for amplification of the full length *SUB* cDNA: GGGGACAAGTTTGTACAAAAAAGCAGGCTTCATGAGCT TTACAAGATGGGAAGTGT and GGGGACCACTTTGTACAAGAAAGCTGGGTCTTAGATCATATGTTGA AGATCTTGG; for the amplification of the ICD version of SUB: GGGGACAAGTTTGTACAAAAAAGCAGGCTTCatgTATAACCGATATTACAGTGGAGC and GGGGACCACTTTGTACAAGAAAGCTGGGTCTTAGATCAT ATGTTGAAGATCTTGG. *AN* cDNA was cloned into pGEX2TMGW (GE Healthcare) to generate an N-terminal fusion with Glutathion-S-Transferase (GST:AN) and the SUB and SUBICD cDNAs were cloned into pETG-40a (EMBL, Heidelberg) to generate an N-terminal fusion with maltose-binding protein (MBP:SUB, MBP:SUBICD).

### Antibody generation

To produce anti-AN antibody, AN was expressed as a GST-fusion protein in *E.coli*. The protein was purified and used to generate antibodies in rabbit (Pineda Antikörper-Service; Berlin, Germany). The antibody serum was affinity purified and checked for its specificity by MALDI-TOF analysis.

### In vitro pull-down assay

Interactions between SUB and AN were studied using purified proteins that were expressed in bacteria. The bacterial cells BL21-CodonPlus (DE3)-RIL containing the IPTG inducible constructs (MBP-Full length SUB, MBP-SUBICD and GST-AN) were grown at 37°C and 220 rpm to an OD 600 of 0.8-0.9 and then the cultures were induced by adding IPTG to final concentration of 1 mM. The induced cells were then grown further for 5 hours (at 37°C for GST-tagged AN constructs and 20°C for MBP-tagged constructs) and cells were harvested by centrifugation. Cells were lysed in Tris-lysis buffer (Tris (pH 7.5) 50 mM, NaCl (100 mM), EDTA (1 mM), EGTA (1 mM), NP-40 (1%), Lysozyme (200 μg/ml), DTT (1 mM), Protease inhibitor cocktail (Sigma)) and sonicated three times for one minute each. The supernatant was collected by centrifugation at 4°C. MBP-tagged proteins were purified by incubation with amylose resin overnight at 4°C. After several washings, part of the resin was boiled with SDS-PAGE gel loading buffer to get purified protein for analysis. The remaining resin was used for incubation with equal amounts of GST:AN lysate for 4 hours at 4°C followed by several washings. Finally, the beads were boiled in SDS-PAGE gel loading buffer at 96°C for 10 min and equal amounts were loaded on a gel followed by western blotting. Detection was done using primary anti-AN antibody and secondary anti-rabbit antibody using SuperSignal West Femto Maximum Sensitivity Substrate (Thermo Scientific).

### Histochemical analysis, microscopy and BFA treatments

Histochemical localization of ß-glucuronidase (GUS) activity in whole-mount tissues was performed as described previously [16]. Scanning Electron Microscopy was made using a Quanta 250 FEG (FEI) microscope under low vacuum conditions without any fixation steps. Confocal laser scanning microscopy and BFA treatments were performed as reported previously [6].

## Competing interests

The authors declare that they have no competing interests.

## Authors’ contributions

LF, MH, KS designed the study. YB, LF, PV and HB performed experiments. YB, LF, PV, HB, MH and KS analyzed the data. MH and KS wrote the manuscript. All authors read and approved the final manuscript.
